# Strain-Rate Dependence of Elastic Modulus Reveals Silver Nanoparticle Induced Cytotoxicity

**DOI:** 10.5772/61328

**Published:** 2015-01-01

**Authors:** Matthew Alexander Caporizzo, Charles M. Roco, Maria Carme Coll Ferrer, Martha E. Grady, Emmabeth Parrish, David M. Eckmann, Russell John Composto

**Affiliations:** 1 Department of Materials Science Engineering, University of Pennsylvania, Pennsylvania, USA; 2 Department of Anesthesiology and Critical Care, University of Pennsylvania, Pennsylvania, USA

**Keywords:** elastic modulus, nano-indentation, VIVA, strain-rate dependent elasticity, dextran, nanogel, silver nanoparticle, silver cytotoxicity, standard linear solid model, cell viscoelasticity

## Abstract

Force-displacement measurements are taken at different rates with an atomic force microscope to assess the correlation between cell health and cell viscoelasticity in THP-1 cells that have been treated with a novel drug carrier. A variable indentation-rate viscoelastic analysis, VIVA, is employed to identify the relaxation time of the cells that are known to exhibit a frequency dependent stiffness. The VIVA agrees with a fluorescent viability assay. This indicates that dextran-lysozyme drug carriers are biocompatible and deliver concentrated toxic material (rhodamine or silver nanoparticles) to the cytoplasm of THP-1 cells. By modelling the frequency dependence of the elastic modulus, the VIVA provides three metrics of cytoplasmic viscoelasticity: a low frequency modulus, a high frequency modulus and viscosity. The signature of cytotoxicity by rhodamine or silver exposure is a frequency independent twofold increase in the elastic modulus and cytoplasmic viscosity, while the cytoskeletal relaxation time remains unchanged. This is consistent with the known toxic mechanism of silver nanoparticles, where metabolic stress causes an increase in the rigidity of the cytoplasm. A variable indentation-rate viscoelastic analysis is presented as a straightforward method to promote the self-consistent comparison between cells. This is paramount to the development of early diagnosis and treatment of disease.

## 1. Introduction

The viscoelasticity of a cell reflects its function in an organism. Migratory cells, such as macrophages and neutrophils, are compliant. In contrast, osteoblasts, which generate bone, are stiff. [1] Interplay between the mechanical properties of cells and their environment is crucial to maintaining homeostasis and viscoelastic changes in cells that are associated with disease. [2–4] In particular, the two leading causes of death in the United States, cardiovascular disease and cancer, [5] progress by mechanical changes in tissue. [3, 6] In the progression of cardiovascular disease, mechanical stiffening of the arterial system, atherosclerosis, can occur by elastin depletion, collagen deposition, endothelial cell dysfunction, hypercholesterolemia or hormonal imbalance. [3] Clinically, the pathological trigger that is responsible for the stiffness change is difficult to pinpoint and atherosclerosis is typically identified by a single symptom – isolated systolic hypertension. [3] Tumours are stiffer than the surrounding tissue, which enables their growth. [4] Meanwhile, the metastatic transformation of malignant cells, which ultimately determines the cancer lethality, is directly associated with the mechanical softening of the cell. [6–8] In particular, the intravasation and extravasation of cancer cells require extensive cytoplasmic deformation. Thus, they are associated with a reduction in both the stiffness and viscosity of the cytoplasm. [4, 6] An atomic force microscopy (AFM) can distinguish metastatic cancer at the single cell level by cell stiffness. [7] Here, AFM is shown to be sensitive to changes in cell state by quantifying multiple viscoelastic parameters, namely, relaxation time and stiffness, by a variable indentation-rate viscoelastic analysis, VIVA. Others have shown similar differences through AFM creep tests. [14–17]

The complex elastic modulus of bulk soft materials, such as gels and tissue, is measured at a macroscopic level using rheometry. [9, 10] To measure the viscoelasticity in single cells, techniques, such as micropipette aspiration, [11] optical trapping [12, 13] and atomic force microscopy, [4, 14–19] combine micrometre to nanometre spatial resolution with piconewton force sensitivity. The indentation of cells using optical trapping is sensitive to very low forces (1–50 pN). By demonstrating that the rate dependence of the cell elastic modulus decreases at lower strain values, optical trap indentation suggests that the membrane may be purely elastic. [12] Creep tests with an atomic force microscope can measure the viscoelasticity of cells. [14–17] Using creep, the cell phenotype is distinguished by viscoelasticity, [14] and cancer progression is linked to a decrease in single-cell stiffness and viscosity. [15, 17] VIVA probes the same viscoelastic parameters as creep tests but offers the advantage of not requiring continuous contact with the cell. It is also more sensitive to faster relaxations.

Changes in keratin [20] and other intermediate filament expressions lead to mechanical property changes that are associated with disease. [21] Within a cell, the degree of actin polymerization is shown to be the primary factor that determines cytoskeletal stiffness and viscosity. [16] The degree of actin polymerization (i.e., f-actin concentration) is linked to metabolism through the ATP/ADP ratio. [22] This suggests that cell stiffness scales inversely with the metabolic rate. Although metabolic changes are a hallmark of diseases, such as cancer, [23] a link between viscoelastic changes and cell metabolism is lacking.

To maximize efficiency, cells compartmentalize their processes. Therefore, viscoelasticity is spatially heterogeneous across cells. For example, the leading edge of a migrating cell exhibits treadmilling of densely branched f-actin, while the f-actin concentration in the cytoplasm decreases. [2] Nuclear viscoelasticity, which is greater than cytoplasmic viscoelasticity, is determined by the organization and expression of a family of proteins that are known as lamins. [24] Cell-cell and cell-matrix adhesions are mediated by the expression of integrins, which are overexpressed in tumour cells. [25] The spectrin family of proteins is responsible for cell shape and membrane pretension, which provides neurons with their elasticity. [26] Consequently, spatially resolved measurements of viscoelasticity in specific areas on individual cells are required to complete the picture of cell health and the ongoing processes that correlate with the onset and progression of disease.

Herein, a scanning probe technique, which is based on an atomic force microscopy, is extended to accurately determine cell viscoelastic parameters with sub-micron spatial resolution. A variable indentation-rate viscoelastic analysis, VIVA, has advantages over force displacement (creep) measurements. This is because indentation curves are used to obtain spatially resolvable frequency sweeps. VIVA is used to detect viscoelastic changes in the cytoplasm of THP-1 cells induced by the toxicity of the silver nanoparticle, Ag NP. Dextran-lysozyme carriers (Dex-Gels) are used to deliver Ag NPs or rhodamine B to the THP-1 cells. In the fluorescent viability assay, rhodamine B and AgNP loaded Dex-Gels show some THP-1 cell toxicity. This correlates with an increase in cell stiffness and viscosity. However, the VIVA shows that there are no changes in the relaxation time of the cells. Furthermore, the ingestion of drug-free Dex-Gels, which show no toxicity in the fluorescent viability assay, does not change the viscoelasticity of the THP-1 cell cytoplasm. This rules out the possibility that the change in viscoelasticity observed with VIVA is simply due to the ingestion of Dex-Gel, and is actually correlative with cytotoxicity. These findings are significant to correlate changes in viscoelasticity with cytotoxicity at the single cellular level using AFM.

## 2. Polyacrylamide Gel Standards: Parallel Plate Rheology vs. VIVA

**Figure 1.**
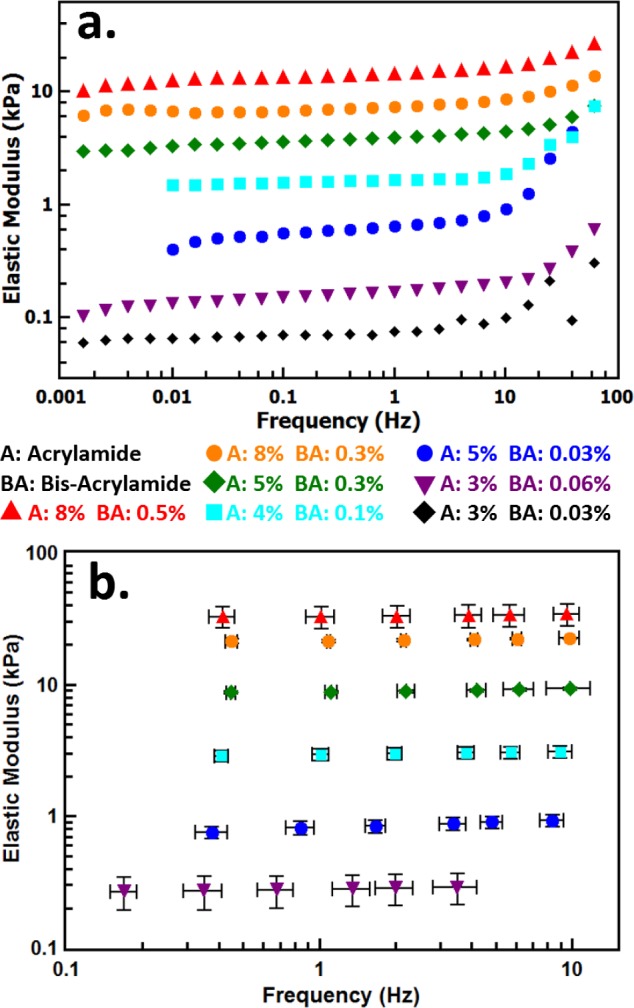
Modulus of Polyacrylamide Gels. a. The bulk shear modulus is determined as a function of frequency by parallel-plate rheology and converted to an elastic modulus using equation 1. b. The elastic modulus of polyacrylamide gels is determined at different frequencies by fitting force-distance curves, which are taken at different rates with an AFM to the Hertz equation (see supplement).

Polyacrylamide gels (PAGs) with varying elastic moduli were synthesized to connect well-established bulk measurements of shear modulus to AFM nano-indentation measurements of elastic modulus. The PAGs were formulated using various combinations of acrylamide and bis-acrylamide concentrations (c.f. caption Figure 1) to generate a range of gels with elastic moduli that span two orders of magnitude in a biologically relevant range (100 Pa – 60 kPa). [27] The elastic modulus (E) that is measured during indentation (compression) is directly related to the shear modulus (G) by Poisson's ratio (ν), which is 0.48 ± 0.12 for PAG. (c.f., equation 1). [28] For all of the tested PAGs, the loss modulus (G‘) is an order of magnitude that is smaller than the storage modulus (G″). This indicates that the gels behave as elastic solids in this frequency range. As G″≪ G′ for PAGs, G″ does not contribute in the conversion of G to E and AFM measurements can be directly compared to bulk rheology using only G′ by

(1) $$E=2G^{\prime }\left(1+\nu \right).$$

Figure 1a shows how the elastic modulus of the PAGs depends on the frequency with a well-established parallel-plate rheology. For all of the PAGs, a plateau of the elastic modulus is observed between 0.1 and 10 Hz. This plateau modulus increases from 25 to 13000 Pa as the gel cross-linking density increases with the acrylamide and bisacrylamide concentration. Over the frequency range that is shown, the PAG moduli do not show a strong strain-rate dependence, which is consistent with their elastic nature in this regime.

The AFM can be used to directly measure the elastic modulus (E) of PAGs by pressing on the surface of the gel with an AFM cantilever and measuring the cantilever deflection as a function of the indentation into the PAG. Deflection can be converted to force using the cantilever spring constant. It can fit to the Hertz equation for the appropriate indenter geometry, i.e., a sphere (discussion in supplement). In AFM nano-indentation, the strain-rate is the velocity at which the cantilever indents the PAG. The velocity of the cantilever, V, divided by the amount of indentation into the PAG, δ, determines the effective indentation rate in Hz. This can be plotted as a compressive analogue to the rheology data shown in Figure 1a.

**Figure 2.**
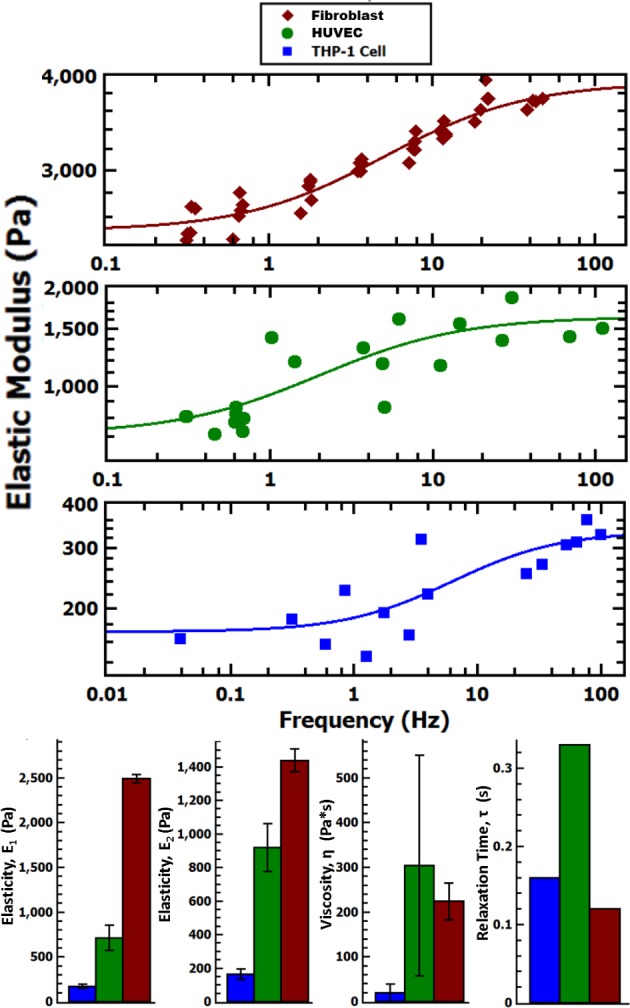
Rate dependence of the cell modulus. The strain-rate dependence of the elastic modulus for three cell types determined by force-distance curves at different rates fit to the Hertz equation. The modulus was measured in the cytosolic region of all three cell types and the solid lines represent fits to the SLSM.

Figure 1b shows a plot of the elastic modulus vs. the strain-rate (V/ δ = Hz) on the PAGs of different cross-linking density. The mean value of the elastic modulus agrees closely with the modulus determined by AFM of the PAGs that were synthesized by the same method as that used in the previous literature. [27] The elastic moduli of the PAGs show little strain-rate dependence for all of the acrylamide and bisacrylamide concentrations tested. Thus, AFM indentation is consistent with rheology for measuring strain-rate dependence (or lack thereof). While the magnitude of the modulus measured by AFM in Figure 2b agrees closely with previous work [27], the bulk rheology measured on the same PAGs consistently estimates a modulus value that is about fourfold lower than the compressive AFM indentations. This may be due to the difference in loading between the shear and compression of the gel, or due to a regular slip of the hydrated gel between the smooth plates of the rheometer. Importantly, the frequency dependence of the elastic modulus is in qualitative agreement between the AFM and rheology. This indicates that the VIVA method does not introduce erroneous frequency dependence in a modulus.

## 3. Cells Exhibit a Strain-rate Dependent Elastic Modulus

The strain-rate dependence of a cell's elastic modulus has been previously established for a number of different cell types. In such studies, indentation at different strain-rates [12] and creep tests [14–17] have been used to show that the viscoelasticity of a cell can be approximated as a standard linear solid. While most indentation-based approaches seek to eliminate hysteresis in the measurement and identify a purely elastic response, this paper aims to utilize the rate dependence of the modulus and test whether these measurements correlate with cytoskeletal dynamics. To determine whether the Hertz model fits to the force-indentation curves yield different elastic moduli at different strain-rates, three cell types were indented at progressively increasing indentation-rates and the corresponding elastic moduli are plotted in Figure 2.

Figure 2 clearly shows that fibroblasts, HUVECs and THP-1 cells exhibit a strain-rate dependent modulus. The fibroblasts were found to be stiffer than the HUVECs. Furthermore, the THP-1 cells were found to be the softest of the three cell types. Independent of cell type, the strain-rate dependence of the elastic modulus was characterized by a plateau in the elastic modulus at a high strain-rate and a finite modulus as the strain-rate approaches 0. This type of modulus dependence on strain-rate is consistent with a standard linear solid model, SLSM. The solid lines in Figure 2 represent the fits of the SLSM model to experimental measurements (see derivation in supplement).

## 4. Using a Standard Linear Solid Approximation to Extract Cell Viscoelasticity

The viscoelastic components of the SLSM are the low frequency modulus, E_1_ the high frequency modulus, E_2_, and the viscosity, η. These parameters can be extracted by fitting the frequency dependence of the cell modulus to the anticipated strain-rate response (c.f. Figure 2). The elastic modulus, *E*, is the transfer function relating stress, $\vec{\sigma }$, to strain, $\vec{e}$, as described in equation 2,

(2) $$\vec{\sigma }=E\vec{e}.$$

For the SLSM, the elastic modulus can be related to the indentation frequency by

(3) $$E=\left(\frac{\eta f\left(E_{1}+E_{2}\right)+E_{1}E_{2}}{E_{2}+\eta f}\right),$$

where the indentation frequency is the ratio of the indentation velocity, *V*, and the indentation depth, δ, *f = V*/δ. This represents the reciprocal of the time over which the force-indentation curve is collected. The experimentally measured elastic modulus, *E*, is the compressive modulus that is determined by fitting the deflection of the AFM cantilever during contact with the cell to the Hertz equation [37] (c.f. supplement), which yields E_1_, E_2_, and *η.* The relaxation time *τ=η/E*_2_, which represents the biomechanical response time of the cell, can also be extracted from the SLSM. In an entangled polymer network, such as the actin cytoskeleton, the relaxation time represents the time for a stress applied to the network to relax by polymer self-diffusion. Inside the cell, the relaxation time may be determined by an ensemble of molecular rearrangements such as the diffusion of actin filaments, dynamic cross-linking of the cytoskeleton, which may or may not slip or release on the time scale of the measurement, membrane elasticity, and other factors which depend on the cell type and cell state.

**Table 1.** Viscoelastic parameters of different individual cells

Cell	E1 (Pa)	E2 (Pa)	η (Pa*s)	τ (s)
**Fibroblast**	2491 ± 46	1437 ± 68	224 ± 41	0.15
**HUVEC**	714 ± 143	918 ± 142	305 ± 246	0.33
**THP-1**	172 ± 25	163 ± 31	19 ± 20	0.12

The relaxation times that are listed in table 1 may depend on the cell type. Namely, the fibroblasts and THP-1 cells exhibit similar relaxation times. This is potentially because these cell types exhibit greater mobility than the HUVEC cells and need to be compliant on timescales that are shorter than the cells or extracellular matrix that they move through. Compared to the THP-1 cells and fibroblasts, the HUVECs appear to have a longer relaxation time. The longer relaxation time of the HUVECs is consistent with their role in the endothelium. Here, they remain anchored and must maintain similar cytoskeletal properties for long periods of time. In response to external stress, the endothelial cells will remain in place longer than the fibroblasts or THP-1 cells. This enables the migratory cells to move through a confluent layer of the HUVECs without dislodging cells.

## 5. Dex-Gel Treated THP-1 Cells

In order to determine whether changes in elastic modulus, viscosity or relaxation time are reflected in changes in cell health, the THP-1 cells are treated with a novel hydrogel carrier (Dex-Gel). This can be impregnated with toxic silver particles. Dex-Gels consist of a lysozyme inner core cross-linked with a dextran coating to form a 150 nm diameter nanoparticle that is digestible, capable of delivering drugs to cells and suitable for grafting specific proteins for targeted cell delivery. [30] Three variations of Dex-Gels are used: (1) blank dextran-lysozyme nanoparticles (Dex-Gel) to test carrier toxicity; (2) rhodamine-labelled dextran-lysozyme particles (Rd Dex-Gel) to track uptake and degradation by fluorescent microscopy; and (3) silver nanoparticle (d = 5 nm) loaded dextran-lysozyme particles (Ag Dex-Gel), which can stress and kill THP-1 cells. The cells were exposed to the Dex-Gels (20 μg/mL) for a 24 hr incubation period. They were then imaged.

**Figure 3.**
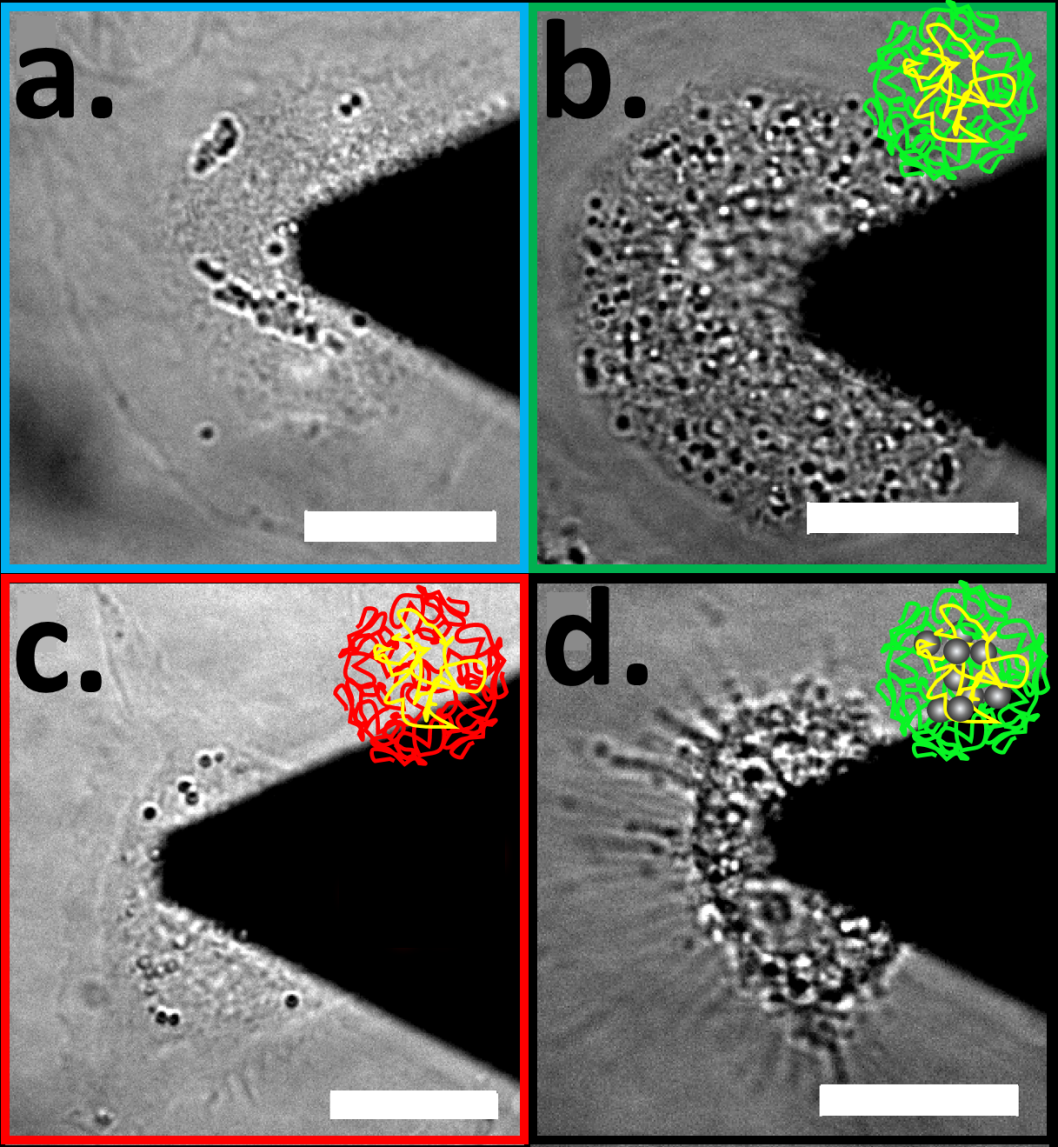
Optical micrographs of the THP-1 cells, which exhibit treatment dependent morphology. a. Untreated THP-1 Cells (control) b. Unloaded lysozyme-dextran conjugate nano-gel drug carriers (Dex-Gel). c. Rhodamine labelled dextran nanogels (Rd Dex-Gel). d. Dex-Gel loaded with 5 nm silver nanoparticles within the core (Ag Dex-Gel). The THP-1 cells are exposed to Ag Dex-Gels 24 h prior to imaging. A schematic of each drug/ carrier complex is drawn inset. Scale bars are 20 μm.

Figure 3 shows optical micrographs of the AFM cantilever (dark triangle) contacting a representative THP-1 cell in each population. Three morphologies are observed: well spread (normal), spoke-like in shape or elongated. In all of the populations, spherical cells that adhere poorly to the coverslip are rare (<5%) and are not counted. The control THP-1 cells (Figure 3a), which are unexposed to the Dex-Gel, are well spread and adhere to the coverslip. The Dex-Gel treated cells (Figure 3b) are similar in morphology to the untreated THP-1 cells. They exhibit the fewest percentage of cells with a spoke-like morphology (14%) and the highest percentage of well spread cells (52%). The Rd Dex-Gel treated cells (Figure 3c) appear to be morphologically similar to the untreated cells, which have a similar number of spoke-like cells (25%). The population of the Ag Dex-Gel treated cells exhibits the largest percentage of spoke-like cells (43%) such as the cell that is shown in Figure 3d (c.f. figure S2).

## 6. THP-1 Cell Mechanical Properties Differ Between Cell Regions

**Figure 4.**
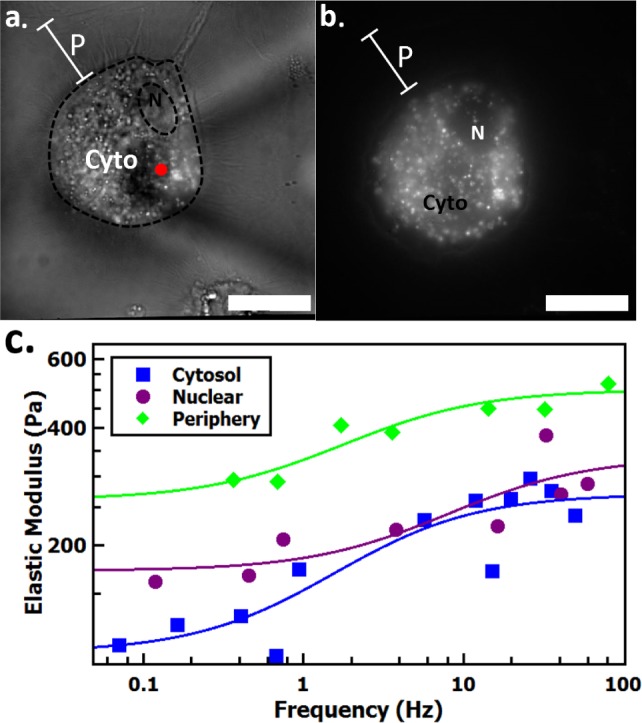
Localization of the Rd Dex-Gel inside the THP-1 cell 24 h after exposure a. Simultaneous white light and fluorescent imaging that show distinguishable components of the cell. The cell periphery, P, cytosol, cyto, and nucleus, N, are labelled. The outline of the retracted AFM cantilever is visible and a red dot is drawn to the approximate size of the microsphere indenter. b. Red fluorescence image of the same cell shows the Rd Dex-Gels (white dots) and diffuse rhodamine (grey cloud) in the cell. The Dex-Gels are localized in the cytoplasmic region (Cyto) c. VIVA to identify the rate dependence of the elastic modulus of P, Cyto, and N of a THP-1 cell modulus results are N=28 measurements from different areas of a single representative HUVEC cell. Fits to SLSM have R^2^ = 0.89, 0.86 and 0.80 for the periphery, nuclear and cytosol respectively. Scale bars 20 μm.

To be certain that self-consistent indentation was performed on a cell-by-cell basis, an inverted optical microscope with resolving capacity, which is sufficient to identify the nucleus, cytosolic region and cell periphery, was integrated with the atomic force microscope. The optical micrographs in figures 4a and 4b show that the THP-1 cells uptake Rd Dex-Gel, which localizes exclusively in the cytosolic region of the cytoplasm (Cyto, Figure 4b). The fluorescent imaging of the THP-1 cells treated with Rd Dex-Gel indicates that all of the cells treated with Dex-Gel uptake a significant amount of the drug carrier and that none of the cells are found to be resistant to the Dex-Gel uptake. The rhodamine fluorescence shows white spots (Rd Dex-Gels) and a cytoplasmic haze, which comes from the dye being released as the Dex-Gels are degraded. Neither the nuclear (N) nor cell periphery (P) regions contain Dex-Gel, while free rhodamine (uniform intensity) penetrates these regions. Taking advantage of the combined optical microscopy and AFM, the nuclear, cytoplasmic and peripheral regions are selectively indented and are found to exhibit different stiffness (Figure 4c).

Figure 4c shows that all three regions of the THP-1 cell show significant strain-rate dependence of the elastic modulus. This dependence is in good agreement (R^2^ > 0.7) with the SLSM (solid traces). The cytoplasmic region is softest at all of the strain-rates and is comparable to the stiffness in the nuclear region. This is likely to be because the indentation probes a combination of stiffness of the nucleus and cytosol in this region. The cytosolic region represents the thicker region of the cell, up to 10 μm, which is optimal for nano-indentation (δ_max_ = 0.5 μm ≪ 10 μm). The periphery is much thinner at circa 1 μm and may appear stiffer due to substrate effects (δ_max_ = 0.5 μm ∼ 1 μm). The periphery is the stiffest region by twofold. All of the further modulus measurements were taken from the cytoplasmic region because this is where the Dex-Gels localize. This area is sufficiently thick for indentation and is wide enough to allow for measurements in multiple areas of the same cell. The nucleus is contained in this cytosolic region and was deliberately avoided to prevent overestimation of the modulus.

## 7. Ag Dex-Gel Treated Cells Are Stressed

**Figure 5.**
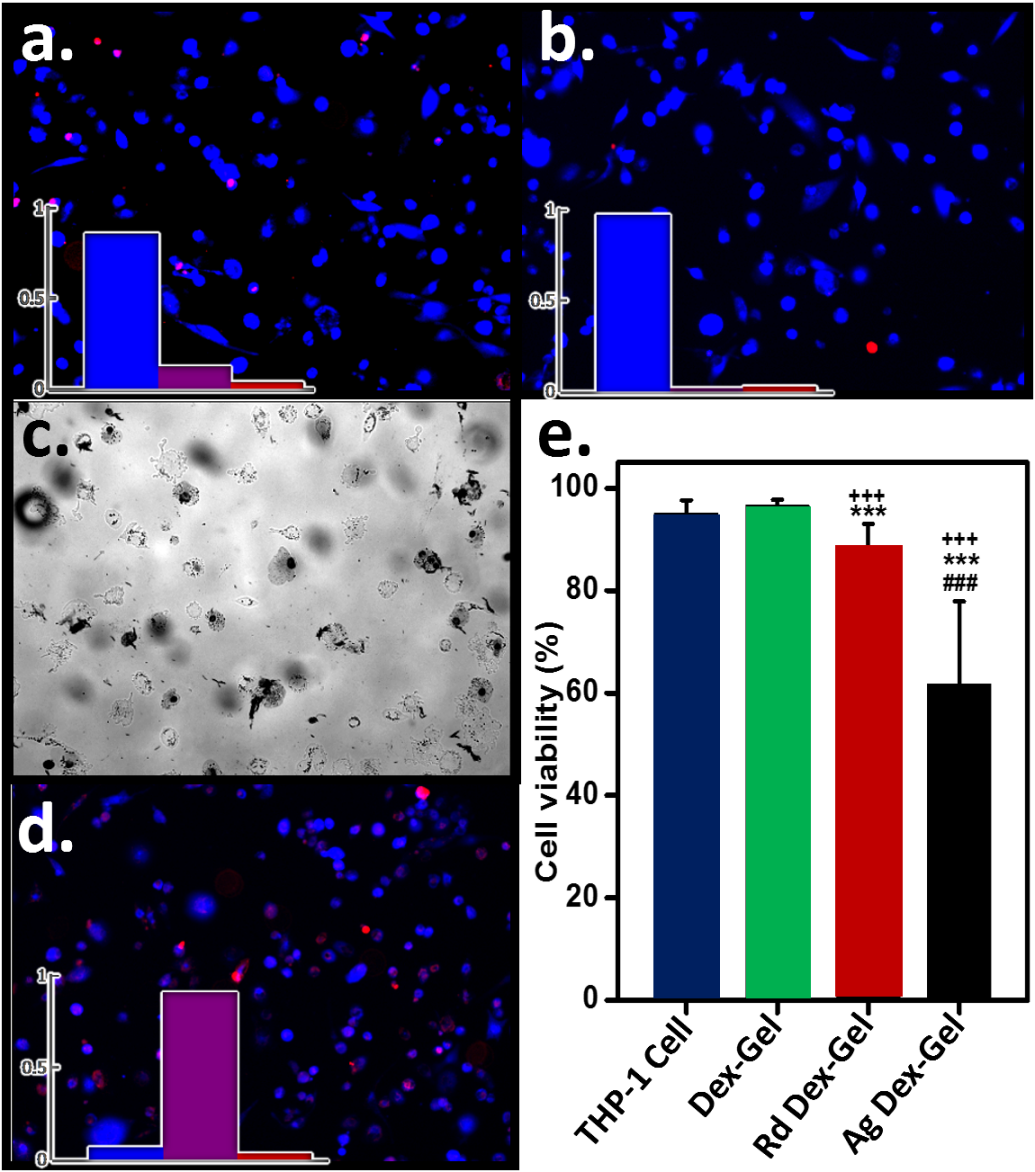
Viability staining of the THP-1 cells 24 h after exposure to the Dex-Gels. a. The untreated THP-1 cells (control) appear predominantly healthy (blue staining dominates histogram) b. The Dex-Gel exposed THP-1 cells are also healthy at 24 h, indicating biocompatibility of the carrier. c. The Rd Dex-Gel loaded THP-1 cells require a different stain due to the fluorescence of the rhodamine dye, thus no histogram indicating stressed or unstressed population can be generated. d. The Ag Dex-Gel loaded THP-1 cells appear to be stressed, as cells expressing both dye (purple) dominate the histogram. e. Viability histogram for all populations. If the intensity of the blue is greater than the intensity of the red dye, a cell is counted as alive. Inset histograms (a, b, and d) show the proportion of each population, which expresses blue, red or both (purple) dyes.

Cell viability studies determine the health of the THP-1 cell populations after 24 h of treatment with the Dex-Gel (Figure 5). Calcein violet (blue) intensity measures the esterase activity inside cells, while the ethidium homodimer (red) penetrates the damaged cell membranes. In Figure 5a-d, the dead cells appear entirely red, the healthy cells (fully active, intact membrane) appear entirely blue, and the injured cells express both dyes. The inset histograms in Figure 5a-d breakdown the dye expression in each cell population by entirely blue, entirely red, or blue and red (purple) cells. The Rd Dex-Gel, Figure 5c, could not be assessed by this method because of the background fluorescence from the rhodamine dye. The control cells and the Dex-Gel treated cells (Figure 5a and 5b, respectively) appear normally active and healthy (high blue intensity and no red intensity). The Ag Dex-Gel treated cells express both dyes, indicating reduced esterase activity and membrane permeability. The staining is consistent with silver inducing injury and stressing THP-1 cells. Silver nanoparticles are known to be toxic to THP-1 cells over exposure times greater than 48 h. Furthermore, silver nanoparticles have been shown to metabolically stress cells by damaging the mitochondrial potential. [31] Thus, it is likely that the Ag Dex-Gel treatment of the THP-1 cells leads to metabolically stressed cells at 24 h, which will eventually (t > 48 h) die of the Ag toxicity. The 24 h exposure time is chosen to specifically probe the viscoelasticity of the THP-1 cells in this stressed state.

Figure 5e shows the dead/alive quantification of the viability assays for the untreated (blue), Dex-Gel (green), Rd Dex-Gel (red) and Ag Dex-Gel (black) THP-1 cells at 24 h. Both the untreated and Dex-Gel treated cells are more than 95% viable. This indicates that the Dex-Gels are non-toxic to the THP-1 cells. The Rd Dex-Gel exposed cells are 90% viable, which is a higher mortality rate than the control. This is consistent with some rhodamine toxicity. The Ag Dex-Gels are 62% viable by the dead/alive quantification method, which suggests substantial toxicity of the Ag Dex-Gels. However, 97% of the Ag Dex-Gel treated cells are injured and still viable by calcein violet staining. The inset histogram (Figure 5d) represents the state of the Ag Dex-Gel population more clearly – only 3% of these cells show no esterase activity (dead), while 92% of the cells express a significant amount of both dyes. Since the majority of the Ag Dex-Gel treated cells are in an injured state at 24 h exposure time, the mechanical properties of the Ag Dex-Gel treated THP-1 cells will be reflective of the toxicity mechanism of Ag nanoparticles.

## 8. AFM Indentation of THP-1 Cells Detects Stiffening in Stressed Cells

Indentation of the THP-1 cells is performed across the relevant frequency range to determine the THP-1 cell viscoelasticity. The minimum and maximum indentation rates are chosen by determining where the modulus becomes independent of the strain-rate. For the THP-1 cells at velocities less than 0.2 μm/s and greater than 10 μm/s, the elastic modulus does not change significantly with the strain-rate. Viscoelastic materials, such as cells, display a maximum in hysteresis at a frequency that corresponds to the relaxation time. Thus, intermediate frequencies are selected by observing where the hysteresis between the indentation and retraction curves on the Dex-Gel treated THP-1 cells is maximized. Using this guideline, two intermediate velocities were selected: 0.5 μm/s and 1.0 μm/ s. Some hysteresis is also observed at the maximum and minimum indentation rates (supplement figure S3). This could be due to the viscoelasticity at these rates or the presence of short-range adhesion between the indenter and the cell. The AFM trigger point is adjusted during the measurement so that the average indentation depth (δ_max_) remains 0.5 μm to avoid any indentation depth dependence of the modulus.

**Figure 6.**
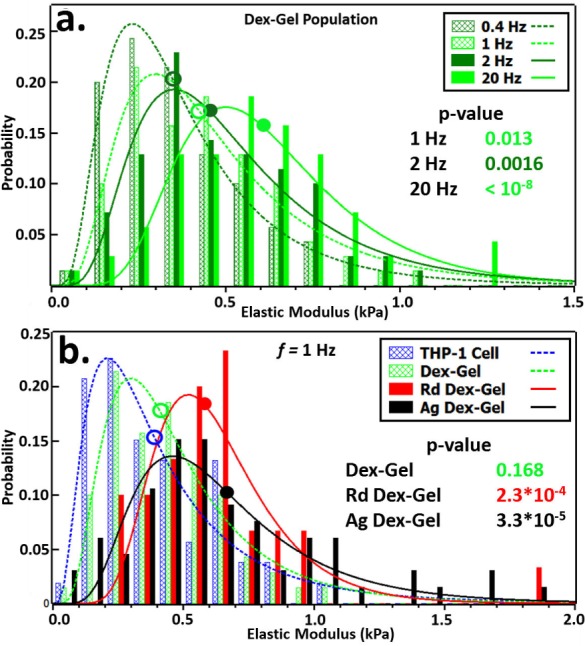
Elastic modulus distribution of THP-1 cell populations. a. Dex-Gel exposed THP-1 cell modulus distributions at four different indentation frequencies. The histograms are well fitted by a log-normal distribution at all frequencies. The circles represent the mean value of distribution. The P-values reflect Mann-Whitney test of distributions, compared to 1 rad/s data. b. The modulus distribution at 3 rad/s indentation frequency of the four cell populations. The histograms are fit by a single log-normal and the dots show the mean value. The P-values reflect Mann-Whitney test of distribution, compared to the THP-1 cell (control). N = 54; Dex-Gel, N=74; Rd Dex-Gel, N=32; Ag Dex-Gel, N = 67. Each modulus measurement (N) at each frequency is the result of between two and five independent indentations.

Figure 6 shows the distribution in the measured elastic moduli for the THP-1 cells and the significance in the difference of the average modulus for the different treatment and measurement conditions. Figure 6a shows the distribution of the Dex-Gel treated THP-1 cell moduli at strain-rates ranging from 0.4 to 20 Hz. The mean cell stiffness increases as the strain-rate increases, while the shape of the distribution remains log-normal. Between the cells, the variance of the modulus is on the order of magnitude of the average modulus. Multiple modulus measurements at one frequency on the same cell exhibit low variance. Therefore, the variance of the modulus distribution is attributed to the viscoelastic differences between the cells and not to the modulus variations within a single cell. The moduli of the Dex-Gel treated population at four different indentation frequencies is well fit by a log-normal distribution with R^2^ > 0.9. The mean of the distribution, which is represented by circles, increases from 351 Pa to 612 Pa, as the indentation frequency increases. The constant high precision of the single log-normal distribution at all frequencies indicates that subpopulations of cells with different frequency responses are not observed.

Figure 6b shows the distribution of the modulus at 1 Hz for the THP-1 cell populations under various treatment conditions. Statistically significant stiffening was observed in the THP-1 cells exposed to the Rd Dex-Gel or Ag Dex-Gels and the stiffening was independent of the frequency (c.f. supplement). The distribution for the untreated THP-1 cells (blue) is comparable to that of the Dex-Gel treated THP-1 cells (green). Both are well fit by a log-normal distribution (blue and green dashed lines) with R^2^ = 0.93 and 0.96, respectively. The average modulus for the untreated and Dex-Gel treated THP-1 cells, which are represented by circles, are similar – 401 Pa and 427 Pa, respectively. The average moduli of the Rd Dex-Gel (608 Pa) and Ag Dex-Gel (689 Pa) treated cells are greater than the control THP-1 cells, p <.001. Although the mean Ag Dex-Gel modulus is greater than the mean Rd Dex-Gel modulus, this difference is not statistically significant, p > 0.1.

A significant broadening in the variance of moduli values for the Ag Dex-Gel THP-1 cells indicates that cells stiffen by different degrees in response to Ag Dex-Gel exposure. In addition, a decrease in the fit quality (table S2) correlates with the increased moduli of the Rd Dex-Gel and Ag Dex-Gel treated cells. This indicates that a stiffer subpopulation may arise, which is not apparent from a visual inspection of the data in Figure 6b. As the quality of fit degrades for the stressed cell populations (Rd and Ag Dex-Gel), the mean of the distribution (circles) no longer correlates with the mean of the log-normal fit and underestimates the actual mean. This deviation is most substantial in the Ag Dex-Gel population, where the mean of the fit is 519 Pa, a 24% underestimate, and the R^2^ value is relatively low, 0.79. Similarly, for the Rd Dex-Gel treated distribution, the fit average is 551 Pa, a 10% underestimate, and the R^2^ is 0.8. While neither the Rd nor Ag Dex-Gel modulus distributions clearly evolve into bimodal distributions, stiff outliers and a reduced R^2^ suggest that anisotropic stiffening gives rise to stiffer subpopulations (c. f. Figure 6b). A correlation between cell stiffening and a degradation of the fit by a single log-normal distribution can be explained by the appearance of a stiffer subpopulation or stiff outliers that are not observed in either of the control groups.

## 9. Viscoelasticity of THP-1 Cells by VIVA

**Figure 7.**
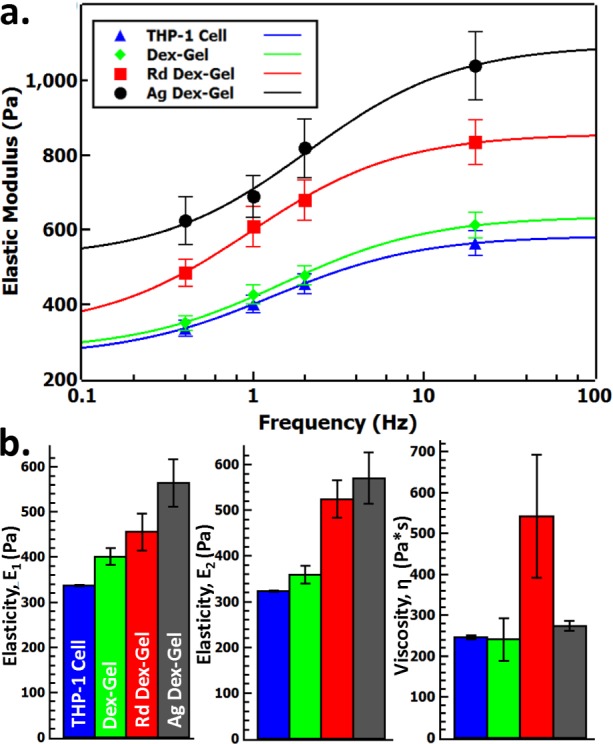
Frequency dependence of the elastic modulus of treated and untreated THP-1 cells. The average elastic modulus determined by nano-indentation of THP-1 cells treated with different Dex-Gels is shown over two orders of magnitude of indentation frequency. The most simple viscoelastic model, which captures the data, is the standard linear solid model (illustrated top). The Ag Dex-Gel and Rd Dex-Gel exposed cells show higher average stiffness (black circles and red squares). The Dex-Gel and untreated cells are routinely softer (green diamonds and blue triangles). Solving the equation of motion for the model shown in the frequency space (top) yields equation 6.4, to which the data are well fitted (solid lines) THP-1 Cell, N = 54; Dex-Gel, N=74; Rd Dex-Gel, N=32; Ag Dex-Gel, N = 67. Each modulus measurement (N) at each frequency is the result of between two and five independent indentations.

In Figure 7, the frequency dependence of an average modulus is shown for all cell populations. As the frequency increases, the elastic modulus of the THP-1 cell increases and approaches a constant value that is near *f* = 20 Hz. At low frequencies, the modulus is also insensitive to frequency. At 0.4 Hz, the modulus value is approximately the zero-frequency elasticity. At intermediate frequencies (between 0.4 and 20 Hz), the transition between the low modulus and high modulus defines the viscosity of the cell. The strain-rate dependence of the elastic modulus is similar for all cell populations. A clear rate-independent increase in the average modulus is observed for the THP-1 cells treated with Rd Dex-Gel and Ag Dex-Gel. Over the range of tested strain-rates, the rate dependence of the THP-1 cells is consistent with the SLSM of viscoelasticity. Creep tests have shown that this is a good model for the stressrelaxation of multiple cell types. [14–17]

Figure 7b summarizes the viscoelastic parameters that are determined by fitting the SLSM to the VIVA data in Figure 7a. The Ag and Rd Dex-Gel treated THP-1 cells exhibit a greater value of E_1_, E_2_, and viscosity compared to the untreated and Dex-Gel treated THP-1 cells. The increase in E_1_ indicates stiffening, which is associated with immobile structures in the cytoplasm or the membrane stiffening. Meanwhile, the increase in E_2_ is indicative of an increase in the cell stiffness. This is associated with macromolecules that are mobile or bonds that release on the timescale longer than the measured relaxation time. The increase in viscosity suggests a reduction in the mobility of the cytoplasmic structures. The relaxation time appears to be conserved in the cell groups that are independent of treatment. This suggests that the mechanism that causes the cells to become stiffer also makes them proportionally more viscous. The scaling of τ may be attributed to the higher cross-linking density of cytoskeletal filaments. This increases the elastic stiffness and restricts the mobility of small molecules in the cytoplasm to flow away from stress (i.e., increasing the viscosity and stiffness together). Furthermore, the E_1_, E_2_, and η values are in good agreement with results from AFM based creep tests. [14–17]

## 10. Physiological Significance of the Strain-rate Dependence

In addition to being spatially heterogeneous, the complexity of the cytoplasm makes it difficult to unequivocally relate the three parameters (E_1_, E_2_, η) directly to physiological processes or cellular components. E_1_ physically represents an entropic spring, which retains its elasticity (does not relax) independent of the timescale. Such stiffness may be attributable to less motile cell contents like the cell membrane, intermediate filament networks and cross-linked filamentous protein networks, which reversibly compress but return the cell to its original shape. The network of filamentous proteins, which is held within the cell membrane, exerts an entropic resistance to deformation. This depends on the length and concentration of the filamentous proteins in the cytoskeleton and could provide the anatomical basis for E_1_. Therefore, a non-zero E_1_ is more sensible than a zero elastic modulus at zero-strain-rate, which would describe a purely viscous cell. Creep tests have also shown the presence of a significant non-zero E_1_. [14–17]

The contribution of elastic component E_2_ occurs when the indentation rate exceeds 1/τ. Thus, it comes from the compression of cytoplasmic materials, such as proteins, molecular motors or cytoskeletal filaments, which, on a slower timescale, are able to flow away from the stress. While the same cytoskeletal structures may influence E_2_, E_2_ remains unique from E_1_. This is because it represents a transient compression of these materials. When the cell is compressed faster than the relaxation time, the cytoskeletal filaments bend or the membrane deforms, which stresses the underlying cortex. On this shorter timescale, the filament cross-links do not have time to slip or release and tension in the cortex cannot relax by an actin filament slip. Thus, the modulus that is felt by the indenter is E_1_ + E_2_ at high frequencies and the mechanical behaviour is elastic. The timeframe η/E_2_ represents the fundamental rate at which these relaxations take place.

Similar values of E_1_, E_2_ and η observed for the untreated and Dex-Gel treated THP-1 cells suggest that the Dex-Gels alone do not alter the cytoskeletal structure and that unloaded Dex-Gels are biocompatible (Figure 5b). The result also indicates that the mechanical properties of the cells are closely linked to their vitality. A false positive for cell stress by AFM (observed stiffening) of this population would indicate that the modulus measurements were sensing the incorporation of the Dex-Gels within the cell. This finding supports the sensitivity of AFM nano-indentation to cell state and its utility as a non-invasive diagnostic tool for evaluating large populations of cells with single cell specificity.

The observed stiffening Ag Dex-Gel and Rd Dex-Gel treated groups is likely to be attributed to the stressed state of these cells. Both the Ag nanoparticles [31] and rhodamine [32] have been associated with injury of the mitochondria, which inhibits ATP regeneration in cells. Rhodamine, in particular, has been shown to change the ATP/ADP ratio in cells enough to change the cytoskeletal structure by increasing the amount of f-actin.[32] Stiffening in stressed cells can be explained by an increase in actin polymerization, which is induced by a slight reduction of ATP/ADP in cells. This may occur in the silver and rhodamine exposed cells, due to either lower mitochondrial potential or ATP and ADP escape through the leaky membrane. [22] An increase in f-actin or even actin filament length would explain the higher measured elastic modulus of the cytoskeleton, which is reflected in the increase of both E_1_ and E_2_.

Filamentous actin has been shown to be the most important cytoskeletal protein in determining the mechanical stiffness in non-muscle cells [16]. Furthermore, an increase in the f-actin expression is consistent with the proposed mechanism of toxicity, which would reduce the ATP/ADP ratio. [22] Thus, this work suggests that metabolic stress can induce mechanical stiffening in cells. This may be consistent with the increased stiffness that is shown in the Rd Dex-Gel treated population (Figure 7) but significantly less toxicity in the viability assay (Figure 5e). Therefore, the stiffening detected by AFM would result in a stressed state in the cell. This occurs as a response to the toxin exposure, which is detectable before the cell is irreversibly damaged. Although beyond the scope of this paper, systematic studies of mitochondrial activity and f-actin concentration in silver and rhodamine stressed cells are needed to firmly establish a link between metabolism and mechanical properties. Such a relationship may be universal. For example, softening is consistently observed in metastatic cancer cells, which are metabolically up-regulated (the Warburg effect), compared to their benign counterpart. [7] Knocking down the actin polymerization by cytochalasin D leads to a tenfold decrease in stiffness, with reductions in E_1_ and E_2_ observed by creep. This is consistent with the idea that the degree of actin polymerization influences these parameters. [16]

Our observations suggest that THP-1 cells exhibit a relaxation time, *η/E*_2_, on the order of 100's of ms. At indentation rates greater than *E*_2_/η, the dynamic components in the cytoskeleton contribute to the elastic modulus, which plateaus as the strain-rate continues to increase. This finding suggests that THP-1 cells are well approximated by a standard linear solid. From the SLSM, the strain-rate dependence of the elastic modulus can be modelled to extract three key parameters that describe cell viscoelasticity: E_1_ is thought to be an indicator of the filamentous actin expression, E_2_ reflects the network cross-linking, and η reflects the changes in the mobility of this network such as the density of cross-links. Future studies are aimed at identifying the biochemical pathways that are associated with biomechanical changes through quantification of mitochondrial potential and the degree of actin polymerization associated with various types of cell stress. Work is also ongoing to extend the frequency range of VIVA so that we can more accurately identify the changes in relaxation times, which represent the fundamental times that are dependent on the biomechanical changes inside the cell.

## 11. Materials and Methods

### 11.1. Nanogel Synthesis

Analytical grade reagents were used, as received. Dextran with a M_w_ = 70 kDa and rhodamine B isothiocycyanate-dextran with M_w_= 64–76 kDa from *Leuconostoc* ssp. were obtained from Fluka Chemie (Buchs, Switzerland) and Sigma-Aldrich (St. Louis, MI, USA), respectively. Lysozyme and silver nitrate (AgNO_3_) were purchased from Sigma-Aldrich. No. 1 glass coverslips (25 × 40 mm^2^) and phosphate buffered saline (without Ca^2+^ and Mg^2+^) were purchased from Fisher Scientific (Hampton, NH, USA).

The dextran-lysozyme conjugate hydrogels, Dex-Gels, are synthesized through a two-step heating process, as previously described. [30, 33] First, the dextran is mixed with lysozyme at a 1:1 ratio in a buffer, and the pH is titrated to 7–8 using 0.1M NaOH. The solution is lyophilized. Next, the amino groups from the lysozyme are reacted with dextran's terminal carbonyl groups in a KBr saturated desiccator at 60°C for 24 h to form the lysosome-dextran conjugates. Once conjugated, gelation is achieved by heating. The conjugate powder is dissolved in water (5 mg/ mL) and the pH is raised to 10.7 (isoelectric point of lysozyme). It is subsequently heated at 80°C for 30 minutes to form a physically cross-linked hydrogel with hydrophobic residue (lysozyme) core and dextran shell. [34] The Dex-Gels are then purified through ultracentrifugation and stored at 4°C.

### 11.2. Rd Dex-Gel

To prepare fluorescently labelled Dex-Gels, rhodamine B isothiocycyanate-and lysosome are mixed in buffer at a 1:1 ratio. The gelation protocol, which is described above, is then followed, yielding similar core-shell Dex-Gels with a rhodamine-B labelled shell.

### 11.3. Ag Dex-Gel

Silver nanoparticles, AgNPs, are grown in Dex-Gels through an additional step following the above procedure to synthesize Dex-Gels. Using 25 mM AgNO_3_ as a precursor, a solution of AgNO_3_ and Dex-Gel is autoclaved to reduce the Ag ions and nucleate AgNPs within the gel. Any outstanding AgNPs are separated from the Dex-Gel solution through a dialysis in deionized water. The subsequent Ag Dex-Gels are roughly 160 nm in diameter and contain 20.4 wt% silver composed of ∼ 5 nm diameter AgNPs. [33]

### 11.4. Polyacrylamide Gel Synthesis

Polyacrylamide gels, PAGs, with defined elastic moduli are formulated using various mixtures of the monomer, acrylamide and the cross-linker, bis-acrylamide, in accordance with two previously set forth protocols. [27, 35] The PAGs are grafted onto an amino-silanated cover slip using a dialdehyde for modulus measurement in phosphate buffered saline, PBS, with an Asylum MFP 3D and built-in analysis software.

Piranha cleaned cover slips are coated with 3-Aminopro-pyltriethoxysilane, APTES, via physical vapour deposition, PVD. The cover slips are then placed in a 0.5% glutaraldehyde solution in PBS for at least 30 minutes. Excess APTES or glutaraldehyde is rinsed off thoroughly with PBS after their respective grafting. Piranha cleaned slides are chloro-silanated through PVD by dichlorodimethylsilane, DCDMS. Solutions containing different percentages of acrylamide and bis-acrylamide in PBS are used to develop PAGs with elastic moduli ratings spanning from 100 Pa to 50 kPa. *N*,*N*,*N*‘,*N*’-tetramethylethylenediamine, TEMED, is added to initiate free radial polymerization, while ammonium persulfate, APS, is added to catalyze the polymerization process. A concentration of 0.1 wt% TEMED and 0.1 wt% of APS are used for all solutions. Once the TEMED and APS are thoroughly mixed, the solution is drop-casted onto a chloro-silanated slide. An amino-silanated cover slip is dropped onto the chloro-silanated slide, creating a “sandwich” effect with the gelling solution in the middle. When polymerization is complete, the chloro-silanated slide can be peeled off, leaving a less than 500 μm thick PAG covalently grafted to the coverslip. The amount of solution drop-casted will affect the thickness of the gel but not the modulus.

PAGs prepared for rheology were drop cast ∼5 mm thick by pouring 1500 μL of polymerizing solution into a 25 mm diameter chloro-silanated glass boat. The gels were then removed from the boat and placed directly onto a RFS3 rheometer with PP25 chuck applying 200 g to the gel. Rheology was performed at 5% strain and 24.1 C between 0.01 and 500 Hz in triplicate. Frequency sweeps at 1% or 3% strain were found to return the same moduli as 5%.

### 11.5. THP-1 Cell Culture

THP-1 cells stably transduced with GFP-actin were maintained in a suspension culture in RPMI media (Gibco, Grand Island, NY, USA), supplemented with 10% FBS (HyClone, Rockford, IL, USA), 1% penicillin streptomycin (Invitrogen, Carlsbad, CA, USA), and 0.05 mM 2-mercaptoethanol. [30,36] For all of the experiments, the cells (13,000 cells/cm^2^) were plated onto glass coverslips (Ted Pella Inc.) in 35 mm polystyrene petri dishes (BD, Ashland, MA, USA). The THP-1 cells were stimulated with the addition of 1 μg/mL of Phorbol 12-myristate 13-acetate, PMA, for 72 h.

### 11.6. HUVEC and Fibroblast Cell Culture

Human vascular endothelial cells were cultured in Vascu-Life VEGF Cell Culture Media (Lifeline Cell Technology, Walkersville, MD) and checked for mycoplasma contamination using MycoAlert Kit (Lonza, Rockland, ME). Adult human dermal fibroblasts (Lifeline Cell Technology, Walkersville, MD) were cultured in Fibrolife cell culture media (Lifeline Cell Technology, Walkersville, MD), as previously dsescribed. [39] The cells were plated onto glass coverslips (Ted Pella Inc.), coated with fibronectin and plated at a density of 5000 cells/cm^2^ in 35 mm polystyrene petri dishes (BD, Ashland, MA, USA). Prior to the experiments, the cells were allowed to grow for 48 h. The AFM analysis was carried out at room temperature (22°C) in HBSS, *p*H 7.4 with 1.3 mmol/l CaCl2, 0.9 mmol/l MgCl2, 2 mmol/l GlutaMax, 10 g/l heparin, 5.6 mmol/l glucose, and 1% FBS) analogous to C. M. Coll-Ferrer et al. [30]

### 11.7. Viability of PMA-stimulated THP-1 Cells

Following stimulation, the THP-1 cells were incubated with the Dex-Gels (20 μg/mL) for 24 h. As a control, the cells were incubated with media. After 24h incubation, the cells were washed with HBSS to remove the Dex-Gel from the solution. The viability of the untreated THP-1 cells, Dex-Gel and Ag Dex-Gel was determined by a fluorescence microscopy. The live cells were stained with calcein violet (750 nM) and the dead cells with ethidium homodimer (2 μM). The viability of the cells incubated with Rd Dex-Gels was determined by an optical microscopy following trypan blue staining (0.4 %). For each condition, five images were collected using a 10 × objective lens. The viable cells were counted using ImageJ (NIH, Bethesda, MD, USA). This experiment was performed in triplicate.

An epifluorescence microscopy was performed using an Olympus IX70 microscope (Olympus, Melville, NY, USA), outfitted with a Chroma Photofluor metal halide light source (89 North, Burlington, VT, USA). Images were captured using a SensiCam QE camera (The Cooke Corp., Romulus, MI, USA) (2 × 2 binning, 688 × 520).IPLab software was used for image acquisition and to control the programmable filter wheels, shutters and focus (Ludl Electronic Products, Hawthorne, NY, USA). ImageJ was used for image analysis.

### 11.8. Instrumentation

An optical microscopy and AFM were performed using an MFP3D AFM (Asylum Research), combined with an inverted Nikon TE-2000U microscope, equipped with a Nikon ×100, 1.49 NA objective. The Rd Dex-Gel fluorescence was captured via a 532 nm laser excitation (Crysta-Laser) and a CCD camera (Cascade-512B, Photometrics) with 200 ms exposure time, controlled through NIS Elements software package. All of the cells were observed with white light illumination from the MFP-3D during modulus characterization. A modulus characterization of the cells was performed using 2 μm diameter SiO_2_ micro-particle functionalized SiN cantilevers with nominal spring constant 0.08 N/m (CP-PNPS-SiO-A, Nano And More, USA). Prior to each experiment, cantilever spring constants were manually calibrated in air using the MFP3D, using a clean glass substrate. The inverse optical lever sensitivity was determined in HBSS solution prior to each round of measurements. The elastic moduli were determined by fitting the indentation curve to the Hertz model, which was adjusted for a spherical indenter using the Asylum Research built-in analysis software. The deflection trigger for each indentation was adjusted prior to each round of measurements to obtain an indentation depth of 500 nm using several sacrificial cells.

## 12. Conclusion: VIVA to Correlate THP-1 Cell Viscoelasticity with Cell Stress

This study utilizes Dex-Gel drug carriers as biocompatible delivery agents of small molecule (rhodamine) and nanoparticle (Ag) toxins to stress THP-1 cells and observe the strain-rate dependent mechanical response by AFM indentation. A fluorescence microscopy shows that the Dex-Gels are incorporated into the cytoplasm of cells, where they are degraded. A viability assay indicates that the unloaded Dex-Gels are biocompatible (>95% vitality at 24 h), while the Ag Dex-Gel treated THP-1 cells become injured in association with the release of Ag in the cytoplasm (Figure 5d). An AFM indentation detects higher stiffness in the Ag Dex-Gels and Rd Dex-Gels, while no stiffness change is observed for the unloaded Dex-Gel exposed cells, compared to the unexposed (control) THP-1 cells (Figure 6b). A variable indentation-rate viscoelastic analysis (VIVA) reveals that the strain-rate dependence of the THP-1 cells is well approximated by a standard linear solid model. It also shows that the cell stiffening in response to cell stress occurs independently of the strain-rate. The relaxation time in the stressed and unstressed THP-1 cells remains protected, while both the low frequency and high frequency modulus plateaus (E_1_ and E1 + E_2_) increase proportionally. Using the SLSM, the VIVA enables three key parameters to be extracted from the indentation-rate dependence of the THP-1 cell elastic modulus: E1 is a measure of immobile elastic structures in the cell, such as the cell membrane rigidity and the f-actin concentration; E_2_ reflects the network cross-linking; and η reflects the mobility of this network related to cross-linking density and biofilament length. These experimental results should facilitate the development of universally comparable cell viscoelasticity models, which can be built up in complexity to further understand the molecular mechanisms that underlie cell biomechanics in health and disease.

## 13. Compliance with Ethical Research Standards

The authors have no conflict of interest to declare. No part of this study was performed on any human or animal subject.

## Figures and Tables

**Figure A1.**
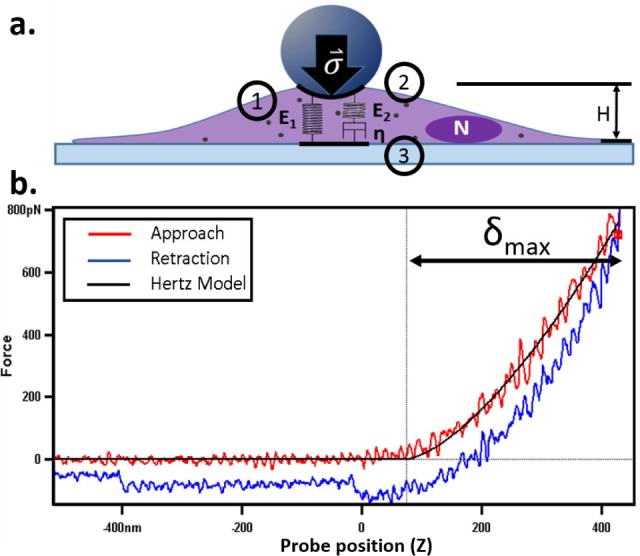
a. Model of SLSM numbering the spring and dashpot elements. The height of the cell, H, nucleus, N, and the stress applied to the cell, σ, are labelled. The elements of the SLSM are numbered so that their corresponding strain response, σ_1–3_ can be catalogued below. b. The typical force-distance curve of a cell fit to the Hertz contact model for a spherical indenter. The maximum indentation depth into the cell, δ_max_, is drawn on the plot. The strain-rate used was 2 μm/s and the trigger point was 10 nm.

**Figure A2.**
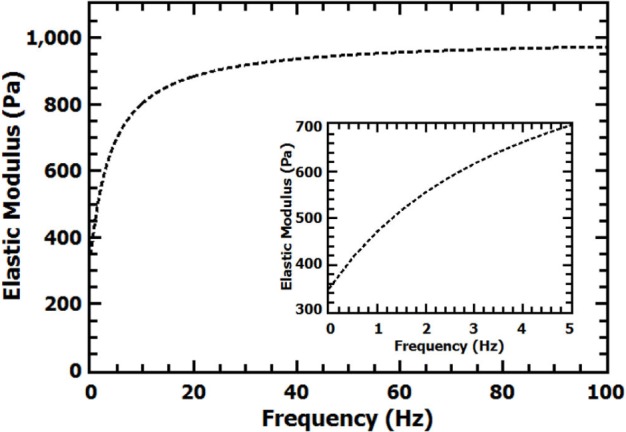
The frequency dependence of the elastic modulus is modelled for a standard linear solid. E_1_ = 350, E_2_ = 640, η = 60.

## Appendix: Derivation of SLSM Based Strain-rate Dependence of Elastic Modulus

The stress-strain response of the individual elements shown in figure A1a are

σ1=E1ϵ=E1δH′σ2=E2ϵ=E2δH andσ3=η∂ϵ∂t=ηδ′H=ηVH .

The general Young's modulus relationship, where the Young's modulus, *E*, is the transfer function between stress and strain.

σ=E=εδH

For the spring dashpot configuration, which is shown in figure A1a, i.e., the standard linear solid model, the stress-strain relationship can be written as a function of indentation depth, δ,

$$\sigma \left(\delta \right)=\sigma_{1}+\frac{\sigma_{2}\sigma_{3}}{\sigma_{2}+\sigma_{3}}.$$

The expression can be expanded for the response of the individual elements and then simplified.

σ(δ)=E1δH+E2δHηδ′HE2δH+ηδ′H==E1δH(E2δH+ηδ′H)+E2ηδ′HδHE2δH+ηδ′H==(δH)(ηδ′δ(E1+E2)+E1E2E2+ηδ′δ)

Extracting strain, = §/H, from the relationship one arrives with

σ(δ)=(δH)(ηδ′δ(E1+E2)+E1E2E2+ηδ′δ)

which has the form σ = *E**ε. Thus,

E=(ηδ′δ(E1+E2)+E1E2E2+ηδ′δ)

and ^δ^/_δ_ has units of s^−1^ and since δ' = V for nanoindentation experiments, it follows that the indentation frequency,

f=δ′δ=Vδmax  ′

where *δ_max_* is the total indentation depth. Therefore,

(4) $$E=\left(\frac{nf\left(E_{1}+E_{2}\right)+E_{1}E_{2}}{E_{2}+nf}\right).$$

The elastic modulus is determined by AFM using the fit of the force-distance curve (figure A1b) to the Hertz equation for a spherical indenter, [37]

$$F\left(\delta \right)=\frac{4}{3}\frac{E}{\left(1-\nu^{2}\right)}R^{1/2}\delta^{3/2}.$$

*R* is the radius of the indenter, δ is the indentation depth, *E* is the elastic modulus and ν is the Poisson ratio. A Poisson ratio of 0.37 was used for this study, as has been previously measured for cells. [38] The solid black line is the best fit of the Hertz equation (above) to the indentation of the cell (red trace). The agreement between the shape of the force-distance curve (red) with the Hertz fit (black) indicates that the Hertz equation is sufficient for determining the elastic modulus of the THP-1 cells. It should also be noted that the Hertz equation is only valid in the low strain limit, where δ ≪ H. This assumption is withheld in these measurements and the general rule of thumb is such that δ < 0.1H. If this is the case, then H can be ignored in the VIVA fit, as it does not appear in equation 3.

The anticipated strain-rate dependence of the SLSM is plotted in figure A2. In analysing the low *f* and high *f* limits of equation 3, the elastic modulus is asymptotic and approaches a value of E_1_ + E_2_ at high frequencies. At low frequencies, the elastic modulus approaches a value of E_1_ (Y-intercept). The corner frequency is the reciprocal of the relaxation time of the material and is related to the viscosity by *f*_c_ = E_2_/η.
